# The Surgical Significance of Vomiting

**Published:** 1908-05-23

**Authors:** 


### THE SURGICAL SIGNIFICANCE OF VOMITING.

### III.—ITS DIAGNOSTIC VALUE.

Much may be done towards elucidating a diagnosis in a case in which vomiting is a symptom by eliciting a careful history of its exact method of onset and its relation to the ingestion of food. For instance, vomiting occurring almost immediately after swallowing should lead one to expect some organic stenosis of the œsophagus; and one may proceed to examine that organ by the passage of an œsophageal bougie, after a thorough examination has made it clear that the symptoms are not due to an aneurysm of the arch of the aorta. Vomiting of this type sometimes occurs where no organic stenosis can be found, the obstacle to the passage of food being a contraction of the œsophagus of functional origin. The condition is rare and only occasionally found in patients of the advanced neurasthenic type. There is, however, one affection of the œsophagus in which vomiting makes its appearance some time after food is taken, namely, a pressure diverticulum. This is a pouch which extends backwards from the œsophagus at its junction with the pharynx, and the explanation of its formation which is usually given is that there is some weakness of the muscular coat in this situation, which allows a slight bulging to take place posteriorly, and that this gradually increases in size owing to the pressure of the food. This explanation does not seem completely satisfactory. It is, at any rate, as reasonable to suppose that the diverticulum is a congenital malformation which exists at birth and may become accidentally opened up during the life of the patient. This view would be analogous to that which is held by some surgeons with regard to the mechanism of a hernia, namely, that a hernia in its descent does not push its peritoneal investment before it, but descends into a pre-existing sac which may have been there since birth, even though the hernia is not of the type which is known clinically as congenital. Whatever the method of formation of these diverticula, they are often associated with the regurgitation of large quantities of food at infrequent intervals. The diverticulum fills up gradually, and when it can hold no more its contents are ejected. The patient can often induce this act at will by pressure on his neck. The ejected material can be differentiated from stomach contents by the absence of an acid reaction and by the fact that it is unaltered by the processes of digestion.

Vomiting is, of course, a constant symptom of diseases of the stomach, since it is the first indication which this organ gives of impairment of its function. It is, for instance, a prominent feature of dyspepsia associated with chronic gastritis. In this case it comes on an hour or so after food, and is accompanied by heartburn and a diffuse pain in the epigastric region. In the chronic gastritis of confirmed alcoholics, on the other hand, there is generally an attack of vomiting in the early morning, in which some slimy mucus is brought up. In a patient suspected of intemperate habits, a question as to his appetite for breakfast will often elicit the answer that so far from having any appetite he is usually sick, and this will tend to confirm one's suspicions, although it is not in itself sufficient evidence to clinch the diagnosis.

If a gastric ulcer be present, vomiting generally comes on, as in gastritis, an hour or so after food; and it may be difficult to distinguish one condition from the other, especially as gastric ulcers of the usual chronic type, are often preceded by chronic dyspepsia, sometimes of years' standing. But in gastric ulcer the epigastric pain is generally more localised, and, of course, the great distinguishing feature is that sooner or later, if a gastric ulcer is present, hæmatemesis will make its appearance. It is true that one sometimes comes across a case of perforation of an acute gastric ulcer where there have been no preliminary symptoms at all, but these cases are fortunately rare—fortunately, because it is almost impossible to arrive at a diagnosis of the cause of the peritonitis in the absence of any data, and in such cases the abdomen is usually opened in the right iliac region in the belief that the case is one of appendicitis.

Gastric dilatation gives rise to a variety of vomiting which is peculiar to itself. The patient vomits large quantities of semi-digested food at intervals which are at first infrequent, but which become shorter as time goes on. It is not uncommon to be told by a patient in the earlier stages that he vomits large quantities once in the twenty-four hours, usually in the early hours of the morning. The cause of this is not far to seek. As the stomach dilates the greater curvature tends to sag down, and there is formed behind the pylorus a pouch, somewhat like that which is formed in the bladder behind a hypertrophied prostate, in which residual urine is retained. In the same way residual stomach contents are retained, and as the organ is unable to get rid of these *per vias naturales*, it does so by regurgitating them. As time goes on the intervals between the acts of vomiting become less and the amount ejected becomes smaller, so that the characteristic nature of the act, as presented in the earlier stages, becomes somewhat obscured. In a case, however, which has been under observation from the outset the diagnosis should be easy, the only difficulty being to arrive at a correct idea of the actual cause of the pyloric stenosis which has led to the gastrectasis. The three main causes which one has to bear in mind are (*i*) a gastric ulcer which has caused a kinking of the pylorus by contracting the stomach, (*ii*) carcinoma of the pylorus, and (*iii*) occlusion of the pylorus by the pressure of a growth in some adjacent viscus. The differential diagnosis of these conditions is often very difficult, especially since a gastric ulcer often forms the starting-point of malignant disease.

One other gastric condition is associated with vomiting of a peculiar type, and that is congenital hypertrophic stenosis of the pylorus. In this the baby ejects the stomach contents with extraordinary force, in some cases, it is said, to such a degree that the stomach contents have been shot over the foot of the cot in which it was lying.

